# Exploring the role of neuronal-enriched extracellular vesicle miR-93 and interoception in major depressive disorder

**DOI:** 10.21203/rs.3.rs-2813878/v1

**Published:** 2023-06-16

**Authors:** Kaiping Burrows, Leandra Figueroa-Hall, Jennifer Stewart, Ahlam Alarbi, Rayus Kuplicki, Bethany Hannafon, Chibing Tan, Victoria Risbrough, Brett McKinney, Rajagopal Ramesh, Teresa Victor, Robin Aupperle, Jonathan Savitz, Kent Teague, Sahib Khalsa, Martin Paulus

**Affiliations:** Laureate Institute for Brain Research; Laureate Institute for Brain Research; Laureate Institute for Brain Research; Laureate Institute for Brain Research; Laureate Institute for Brain Research; Univeristy of Tulsa; Univeristy of Tulsa; Univeristy of Tulsa; Univeristy of Tulsa; Laureate Institute for Brain Research; Laureate Institute for Brain Research; Laureate Institute for Brain Research; Laureate Institute for Brain Research; University of Oklahoma School of Community Medicine; Laureate Institute for Brain Research; Laureate Institute for Brain Research, Tulsa, USA

**Keywords:** depression, exosomes, interoceptive awareness, IL-1, IL-6, TNF, inflammation

## Abstract

Major depressive disorder (MDD) is associated with interoceptive processing dysfunctions, but the molecular mechanisms underlying this dysfunction are poorly understood. This study combined brain Neuronal-Enriched Extracellular Vesicle (NEEV) technology and serum markers of inflammation and metabolism with Functional Magnetic Resonance Imaging (fMRI) to identify the contribution of gene regulatory pathways, in particular micro-RNA (miR) 93, to interoceptive dysfunction in MDD. Individuals with MDD (*n* = 44) and healthy comparisons (HC; *n* = 35) provided blood samples and completed an interoceptive attention task during fMRI. EVs were separated from plasma using a precipitation method. NEEVs were enriched by magnetic streptavidin bead immunocapture utilizing a neural adhesion marker (CD171) biotinylated antibody. NEEV specificities were confirmed by ow cytometry, western blot, particle size analyzer, and transmission electron microscopy. NEEV small RNAs were purified and sequenced. Results showed that: (1) MDD exhibited lower NEEV miR-93 expression than HC; (2) within MDD but not HC, those individuals with the lowest NEEV miR-93 expression had the highest serum concentrations of interleukin (IL)-1 receptor antagonist, IL-6, tumor necrosis factor, and leptin; and (3) within HC but not MDD, those participants with the highest miR-93 expression showed the strongest bilateral dorsal mid-insula activation. Since miR-93 is regulated by stress and affects epigenetic modulation by chromatin reorganization, these results suggest that healthy individuals but not MDD participants show an adaptive epigenetic regulation of insular function during interoceptive processing. Future investigations will need to delineate how specific internal and external environmental conditions contribute to miR-93 expression in MDD and what molecular mechanisms alter brain responsivity to body-relevant signals.

## 1. Introduction

Interoception refers to the nervous system’s ability to sense, interpret, and integrate internal bodily signals across conscious and unconscious levels^1^. Individuals with major depressive disorder (MDD) often report dysregulated interoceptive awareness, or abnormal experiences of their internal body states^2–5^. It has been argued that, within the context of depression, the brain is rigidly insensitive to interoceptive signals and as a result fails to efficiently predict and regulate the body’s metabolic needs^6^. The insular cortex likely integrates interoceptive signals with emotionally salient information during conscious as well as unconscious comparisons of anticipated versus experienced bodily states, a process which relies on the management of interoceptive prediction and prediction-error signals, embodied emotional signals, and self-related signals^7–9^. Moreover, a recent functional magnetic resonance imaging (fMRI) meta-analysis identified blunted activity of the left dorsal mid-insula in patients with MDD relative to healthy individuals across interoceptive awareness paradigms, suggesting that disrupted mid-insular activation may represent a neural marker and a putative target for novel interventions in depression^10^.

Although the cellular and molecular processes resulting in altered interoception in MDD are only beginning to be understood^11^, several hypotheses have been advanced. Among them is the notion that elevated levels of pro-inflammatory cytokines in some depressed individuals result in a decoupling of afferent interoceptive input from interoceptive predictions, leading to increased prediction-error signals^12–14^. Dysregulation of both the innate and adaptive immune systems is consistently described in depressed patients^15^ and increased peripheral inflammatory markers have been linked to disturbed brain function in regions including the insula^16^. In addition to immuno-inflammatory activation, neuroendocrine regulators of energy metabolism such as leptin and insulin are involved in homeostatic adjustments and brain circuits integrating homeostatic and mood regulatory responses^17^. Leptin, a peptide hormone with pro-inflammatory properties that functions to maintain energy homeostasis^18^, can interact with neural circuitry to increase the likelihood of developing MDD^19^. These findings suggest that mechanistic alterations of interoception in MDD may be related to elevated pro-inflammatory cytokine levels, immune system dysregulation, and disturbances in insula activity, as well as involvement of neuroendocrine regulators of energy metabolism and mood regulation.

Measuring neuronal-enriched extracellular vesicles (NEEVs) enables one to examine non-invasively specific molecular processes in the brain. These molecular findings can be integrated with systems-level signals such as fMRI to gain a better understanding of the biological dysfunction related to specific cognitive or affective processes. Extracellular vesicles (EVs), including exosomes (biogenesis occurring through multivesicular bodies inside the cell; size range ~ 40nm to 160nm) and ectosomes (vesicles that pinch off the surface of the plasma membrane by outward budding; size range ~ 50nm to 1μm in diameter) are released by many cell types and can be associated with immune responses, among others^20^. EVs contain proteins, metabolites, and nucleic acids that can be delivered into recipient cells for intercellular communication, thereby effectively altering their biological responses related to regulation of central and peripheral immunity, and/or metabolic reprogramming^20^. Moreover, NEEVs cross the blood-brain barrier from both directions and are involved in central nervous system (CNS) regulation by micro ribonucleic acid (RNA) (MiR) transmission. MiRs are a class of small non-coding RNAs functioning as key post-transcriptional regulators of gene expression through the destabilization of messenger RNA (mRNA); MiRs are enriched in the CNS and more distinct MiRs are expressed in the brain than in any other tissue^21^. Neuronal MiRs account for 70% of all MiRs in our body that are involved in regulating neurogenesis and neuroplasticity^22^. It is important to note that MiRs carried from the brain may enter into blood circulation during major depressive episodes^23^. Identifying MiRs related to neuronal development, inflammation, and metabolic pathways can elucidate body-brain connections and how they differ as a function of MDD presence versus absence.

One such MiR, microRNA-93–5p (miR-93), is a member of the miR-106b-25 cluster, located on chromosome 5^24^, and has been implicated in the regulation of neural progenitor cell proliferation and neuronal differentiation^25^. miR-93 can regulate neurogenesis and neuronal growth^26^ and target mRNAs involved in metabolic signaling^27^. miR-93 reduces inflammatory cytokine expression, including interleukin (IL)-1 beta (IL-1β), tumor necrosis factor (TNF), and IL-6 though signal transduction/activation of the transcription 3 (STAT3) signaling pathway^28, 29^. Another pathway regulated by miR-93, the toll-like receptor 4 (TLR4) inflammatory pathway, has been postulated as one of the key players implicated in the increased inflammatory response in depressed individuals^30^. For instance, research indicates that miR-93 regulates the TLR4/NF-kB pathway^31, 32^ and EV-derived miR-93 protects lipopolysaccharide (LPS)-induced cell injury by inhibiting TNF activation^33^. There is also some evidence showing the link between dysregulated miR-93 and other processes perturbed in depression such as insulin resistance^34, 35^, adipogenesis^36^, which could make it a therapeutic target for obesity and the metabolic syndrome^37, 38^, and chromatin remodeling^39, 40^.

Given the roles of miR-93 in regulating neuronal axogenesis, inflammation, and metabolism, the current study aimed to link these molecular processes to previously described mid-insula dysfunction during interoceptive processing in depression, by integrating brain fMRI and NEEV technology. A sub-sample of MDD and healthy comparison (HC) participants from the Tulsa 1000 (T1000) project^41^ completed an interoceptive awareness task during fMRI and provided blood for isolation and enrichment of NEEVs and immunoassays. We evaluated the role of miR-93 to investigate whether MDD patients differed from HC in: (1) NEEV miR-93 expression; (2) the relationship between NEEV miR-93 expression and inflammatory and metabolic markers; and (3) the relationship between NEEV miR-93 expression and brain activity during interoceptive versus exteroceptive attention.

## 2. Materials and Methods

### 2.1 Participants

Participants in this study were drawn from the first 500 individuals who completed baseline assessments as part of the T1000 project, a naturalistic longitudinal study of 1000 individuals including healthy and treatment-seeking individuals with mood, anxiety, substance use and eating disorders^41^. The T1000 study was conducted at the Laureate Institute for Psychiatric Clinic and Hospital, other local mental health providers, and the general community through radio Brain Research (LIBR) in Tulsa, Oklahoma, United States. Baseline assessments occurred between 1/01/2015 and 12/21/2017. The T1000 project was approved by the Western Institutional Review Board and performed in accordance with the Declaration of Helsinki (ClinicalTrials.gov identifier: NCT02450240, “Latent Structure of Multi-level Assessments and Predictors of Outcomes in Psychiatric Disorders”). Participants provided written informed consent and received compensation for their participation.

Participants were recruited from the Laureate, online, newspaper, and other media advertisements in Tulsa and the surrounding regions of Oklahoma. Participants were evaluated for Diagnostic and Statistical Manual of Mental Disorders–IV or -V (DSM-4 or 5) diagnoses determined by the Mini International Neuropsychiatric Inventory (MINI)^42^. All participants in the MDD groups entered the T1000 study with significant depressive symptoms (the Patient Health Questionnaire (PHQ-9) ≥ 10^43^) and met DSM criteria for a past and/or current MDD diagnosis. See Victor et al and colleagues for complete sample size, demographic and screening measures^41^.

To determine whether individuals with depression show evidence for altered cellular processing related to interoception, only MDD and HC subjects were included in the present analysis. Our previous study using participants from this sample suggested that there was no statistically significant evidence for blood oxygen level-dependent (BOLD) signal differences between un-medicated MDD and MDD with use of selective serotonin reuptake inhibitors (SSRI) on the VIA task^44^; therefore, both unmedicated and SSRI-medicated subjects were included in this analysis. MDD subjects taking selective norepinephrine reuptake inhibitors (SNRI), or taking various other antidepressants were excluded from the current analysis. Participants were also excluded if they had inflammation or metabolic related disease (e.g., autoimmune disease, inflammatory bowel disease, or diabetes), or they were taking anti-inflammatory or anti-diabetic drugs. In addition, subjects were excluded if they had poor quality or missing VIA fMRI data. Finally, 41 MDD and 35 HC participants remained for data analysis (see Table 1).

The following demographic and clinical ratings were assessed: (1) age, sex, education, employment status, International Physical Activity Questionnaire (IPAQ), and body mass index (BMI) metrics; and (2) the Patient-Reported Outcomes Measurement Information System (PROMIS)^45^ depression scales.

### 2.2 Neuronal-enriched EV.

See Supplemental Methods. EV Track ID#EV210507.

#### 2.2.1 Blood collection.

Venous blood was collected during the baseline assessment in BD Vacutainer EDTA Blood Collection tubes then transported to the University of Oklahoma Integrative Immunology Center (IIC) within two hours of collection. Blood tubes were centrifuged at 1300 *× g* for 10 minutes (min) at room temperature (RT), plasma was removed, aliquoted, and then stored at −80°C until analysis.

#### 2.2.2 EV separation.

EV separation method was adapted from our previous publication (Burrows et al 2022b). Plasma was thawed on ice, then centrifuged at 3,000 x revolutions per minute (rpm) for 15 min. 3.5 microliters (mL) of Puri ed Thrombin (500 U/mL) (System Biosciences, CA, United States; Catalog # TMEXO-1) was added to 350 mL of plasma to make a final concentration 5 U/mL. After incubating plasma/thrombin for 5 min at RT and centrifuging plasma/thrombin at 10,000 rpm at RT for 5 min, 300 mL of plasma was used for EV separation. Briefly, 76 mL of ExoQuick Exosome Precipitation Solution (System Biosciences, CA, United States; Catalog # EXOQ5A-1) was added to 300 mL of plasma, incubated 30 min on ice, and then centrifuged at 1,500 *× g* at 4°C for 30 min. After removing supernatants, the pellets were centrifuged at 1,500 *× g* at 4°C for an additional 5 min to remove all traces of supernatant. EV pellets were re-suspended in 300 mL of 1X phosphate buffered saline (PBS) (Thermo Fisher Scientific, United States; Catalog #AM9625) with Halt protease and EDTA-free phosphatase inhibitor cocktail (Thermo Fisher Scientific, United States; Catalog #78425). Finally, 150 mL of EV were used for immunochemical enrichment, and the remaining EV aliquots were stored at −80°C for future analysis.

#### 2.2.3 NEEV enrichment.

EVs were enriched via a magnetic streptavidin bead immunocapture kit targeting the neural adhesion marker, L1CAM (CD171) biotinylated antibody-schematic carton of the NEEV enrichment (see *Burrows et al*^46^). This method for enriching NEEVs in blood samples has been validated^46–49^. The CD171 (L1CAM, neural adhesion protein) marker was used for NEEV enrichment due to its high and relatively specific expression in neurons and low levels of expression in many other cell types^47^.

#### 2.2.4 Flow Cytometry.

Once NEEVs were captured and stabilized, the bead/antibody/EV complex was coupled to the EV marker – CD63 fluorescein isothiocyanate (FITC) and neuronal marker – CD171 Allophycocyanin (APC) fluorescent tags and subsequently analyzed by flow cytometry to con rm EV capture and NEEV enrichment.

#### 2.2.5 Western Blot.

EVs, NEEVs, EV-depleted plasma, total EV after enrichment, and cells were denatured directly in a 4X Laemmli sample loading buffer and separated by SDS-PAGE using Mini PROTEAN^®^ TGX^™^ precast gels (Bio-Rad, Catalog # 4561044). Separated proteins were transferred unto polyvinylidene difluoride (PVDF) membranes using a Trans-Blot^®^ Turbo transfer system (Bio-Rad, Catalog # 1704156). Primary antibodies used include CD171 (1:1000, Invitrogen, Catalog # 13–1719-82), CD81 (1:1000, Santa Cruz, Catalog # SC-166029), Alix (1:1000, Santa Cruz, Catalog # SC-53540), and calnexin (1:1000, Cell Signaling, Catalog # 2679).

#### 2.2.6 Transmembrane electron microscopy (TEM).

For EM, EV samples suspended in water were fixed in 2% paraformaldehyde. Fixed samples were absorbed onto formvar coated copper grids for 20 min. Samples were then fixed in 1% glutaraldehyde for 5 min. After being rinsed in distilled water, samples were stained with 2% uranyl acetate for 1 min. Excess liquid was wicked off the grid using filter paper, and grids were stored at room temperature until imaging. Imaging was performed on a Hitachi H7600 microscope equipped with an AMT NanoSprint 1200 camera at the Oklahoma Medical Research Foundation (OMRF) imaging core.

#### 2.2.7 Particle size and concentration analysis.

The particle concentration and size of EVs and NEEVs were measured using micro fluidic resistive pulse sensing (MRPS) method with the Spectradyne nCS1^™^ instrument (Spectradyne Particle Analysis, Signal Hill, CA, USA).

#### 2.2.8 miRNA Purification.

Qiagen miRNeasy Micro Kit (QIAGEN, United States; Catalog #217084) was used for purification of total RNA including miRNA from EVs and NEEVs according to the manufacturer’s protocol. Small RNA concentration was measured using an Agilent Small RNA Kit (Agilent, United States; Catalog #5067–1548) on a Bioanalyzer 2100 instrument (Agilent, United States). RNA samples were stored at −80°C until sequencing.

#### 2.2.9 miRNA sequencing and data processing.

RNA samples were sent to the OMRF Clinical Genomics Center for Next Generation Sequencing (NGS). Briefly, miRNA libraries were generated with a Qiagen QIAseq MiR library preparation kit and NGS was performed on an Illumina NextSeq HO SR75. Raw sequence FASTQ files received from OMRF were imported to the Partek Flow Software for data analysis. Adapters from 3’ end were trimmed from the raw reads after a quality check, bases trimmed from both ends, and then aligned to the human genome hg38 using Bowtie alignment. Next, the aligned reads were quantified against the human miRbase mature microRNAs version 22 and reads from miR genes were normalized and scaled to reads per million for statistical data analysis.

### 2.3 Immunoassays

Serum interleukin 1 receptor antagonist (IL-1ra) concentrations were measured with Human IL-1ra/IL-1F3 Quantikine ELISA kits (R & D Systems, Minneapolis, USA). Serum TNF and IL-6 concentrations were measured with Proinflammatory Panel 1 Human Kit (Meso Scale Diagnostics, Maryland, USA), and C-reactive protein (CRP) was analyzed with Vascular Injury Panel 1 Human Kit (Meso Scale Diagnostics, Maryland, USA). Human Leptin, Insulin Kit (Meso Scale Diagnostics, Maryland, USA) was used to analyze serum leptin concentrations. All serum samples were tested in duplicate. The intra- and inter-assay coefficients of variation (CV) were 3.1% and 15.6% (IL-1ra), 4.2% and 7.0% (IL-6), 3.1% and 12.1% (TNF-a), 2.5% and 10.0% (CRP), and 6.6% and 8.9% (leptin), respectively.

### 2.4 Neuroimaging

Each participant completed a structural MRI scan followed by fMRI scanning while performing an interoceptive awareness task.

#### 2.4.1 MRI acquisition

MRI images were acquired on two identical General Electric Discovery MR750 (GE Healthcare, Milwaukee, WI) whole-body 3-Tesla MRI scanners. The structural scan was acquired using a T1-weighted magnetization-prepared rapid gradient-echo (MPRAGE) sequence. Anatomical imaging parameters were repetition time (TR)/echo time (TE) = 5/2.012 ms, field of view (FOV) = 240, 186 axial slices, 0.9 mm slice thickness, 256 × 256 matrix, voxel volume = 0.938 × 0.938 × 0.9 mm^3^, flip angle = 8°, acceleration factor R = 2, inversion time = 725 ms. A single-shot gradient-recalled echo-planar imaging (EPI) sequence with Sensitivity Encoding (SENSE) depicting BOLD contrast was used for functional scans. Functional imaging parameters were TR/TE = 2000/27 ms, FOV/slice = 240/2.9 mm, 128 × 128 acquisition matrix, 39 axial slices, 180 TRs, flip angle = 78°, SENSE acceleration factor R = 2 in anterior-posterior direction, and voxel volume = 1.875 × 1.875 × 2.9 mm^3^.

#### 2.4.2 Interoceptive awareness task

The Visceral Interoceptive Attention (VIA) task was comprised of two eight-minute runs, each containing interoceptive and exteroceptive conditions. During the interoceptive conditions, the words “heart” and “stomach” cued participants to attend to sensations from that part of the body. During the exteroception (i.e., control) condition, participants attended to the word “target” as it alternated between black and varying shades of grey. Trials lasted 10 seconds, and half of trials were followed by a 5-second period for participants to rate stimulus intensity (0 = ‘no sensation’ to 6 = ‘extreme sensation’). Each run included 6 trials per condition (intertrial interval range 2.5–12.5 s). The VIA task has been previously shown to be effective at mapping the neural signal associated with interoceptive attention, including in depressed individuals^5, 50–54^.

#### 2.4.3 fMRI data preprocessing

Single-subject image pre-processing was performed using Analysis of Functional NeuroImages (AFNI) software (http://afni.nimh.nih.gov/afni)^55^. The anatomical scan was registered to the first volume of the EPI time-course and then aligned to Montreal Neurological Institute (MNI) space via affine transformation, saving the transformation parameters for application to the EPI data. The first three TRs were discarded from each EPI time-course to allow the fMRI signal to reach steady state, followed by despiking; slice-timing correction and co-registration to anatomical volumes. Motion correction and spatial transformation to MNI space of the EPI data were implemented in a single transformation. The EPI data were then smoothed with a 4mm Gaussian full-width at half-max smoothing kernel, and signal intensity normalized to reflect percent signal change from each voxel’s mean intensity across the time-course. All images were resampled to 2 × 2 × 2 mm^3^ isometric voxels.

#### 2.4.4 Subject-level fMRI imaging analysis

Each subject’s functional imaging data were analyzed using a voxelwise general linear model analysis. Block regressors were convolved with a canonical hemodynamic response function and used to model BOLD responses for heart, stomach, and target conditions. Six motion parameters (three translations and three rotations) were included as nuisance regressors. Censoring was done at the regression step by removing volumes with either a Euclidean norm of the derivatives of the six motion parameters greater than 0.3 mm or greater than 10% outlier voxels, determined by 3dToutcount. Percent signal change during each condition was defined as the estimated beta coefficient from single-subject analysis.

### 2.5 Statistical analysis

#### 2.5.1 Demographic characteristics and clinical ratings

Independent sample t-tests examined differences between MDD and HC on age, sex, IPAQ exercise MET-minutes per week, BMI, and PROMIS depression. Chî2 test was used to access sex differences between groups.

#### 2.5.2 NEEV miRNA analysis

Statistical analyses on NEEV miR-93 were conducted in R. Scaled miR-93 data (counts per million) were log-transformed due to their non-Gaussian distributions determined by Shapiro-Wilks tests. Outliers were defined as *z* = ±3 across subjects and set as missing. Independent sample t-tests were used to assess differences between MDD and HC, as well as between un-medicated and SSRI-medicated MDD subjects. In addition, miR-9, a neuronal cell-specific marker^56^ was compared between NEEV and EV.

#### 2.5.2 Relationship between NEEV miR-93 expression and inflammatory/metabolic markers

All inflammatory/metabolic markers (IL-1ra, TNF, IL-6, CRP, and leptin) were log-transformed due to their non-Gaussian distributions determined by Shapiro-Wilks tests. Outliers were defined as *z* = ±3 across subjects and set as missing. Independent t-test was used to test the group difference on IL-1ra, IL-6, and TNF; relationships between NEEV miR-93 expression and IL-1ra, IL-6, and TNF within each group were tested by Pearson’s correlations.

Even after log-transformation, the distributions for CRP and leptin were found to be non-Gaussian; therefore, Spearman’s correlations were used to test their relationships to NEEV miR-93 expression within each group, and group differences on these two markers were tested using Mann-Whitney-Wilcoxon non-parametric tests. ANOVA tests were used to evaluate slope differences between MDD and HC groups.

#### 2.5.4 Group-level fMRI imaging analysis

AFNI’s 3dttest++ was used to assess the whole brain voxel-wise group by NEEV miR-93 interaction on BOLD activation of the interoception versus exteroception contrast. The group statistical map was corrected for multiple comparisons according to our previous neuroimaging approaches with this task (see Supplemental Methods). BOLD activation of the interoception versus exteroception contrast within clusters with significant group*miR-93 effects were extracted for follow-up analyses. Robust regression tested the slope of different relationships between NEEV miR-93 and BOLD for each significant cluster. False Discovery Rate correction for multiple comparisons was used across the resulting tests.

## 3. Results

### 3.1 Demographics and clinical characteristics

The groups did not differ on age, sex, education, or employment status (Table 1). The MDD group reported lower physical activity as well as higher BMI and PROMIS depression scores than the HC group.

### 3.2 Characterization of EV and NEEV

**Figure 1:**
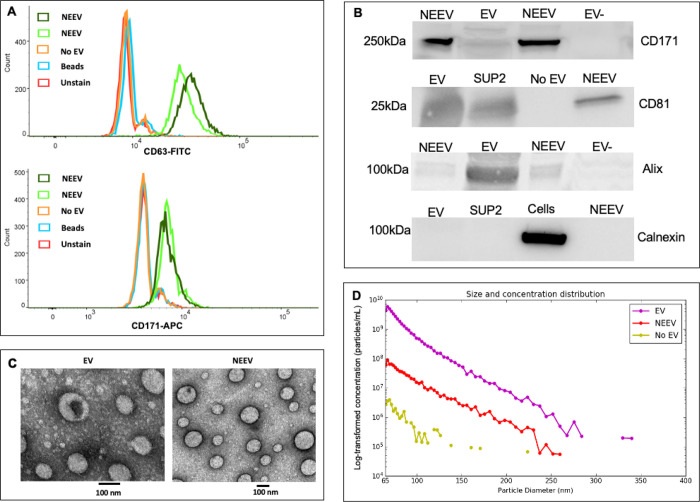
Characterization of EV and NEEV. **A.** Flow cytometry results of neuronal-enriched extracellular vesicles (NEEVs), negative control (No EV), beads and unstained samples with the extracellular vesicle (EV) marker CD63-FITC and NEEV marker CD171-APC. NEEVs were positive for EV marker CD63-FITC and NEEV marker CD171-APC compared to EV-negative control, beads, or unstained samples, as expected. **B** shows western blot analysis of NEEV, EV, and EV negative or positive control with NEEV surface marker CD171, EV surface marker CD81, EV internal marker Alix, and EV negative marker, Calnexin. Note, EV-: EV-depleted plasma; SUP2: total EV after enrichment; No EV: PBS instead of total EV were used for enrichment. Western blot analysis showed that (1) CD171 marker was enriched in NEEVs; (2) EV surface marker CD81 and EV internal marker Alix were present in EVs and NEEVs; and (3) EV negative marker Calnexin was not observed in EV or NEEV samples. **C** Images of EVs and NEEVs with transmission electron microscopy (TEM); images denote a scale bar of 100 nm. **D** depicts size and concentration analysis of EV, NEEV, and EV-depleted plasma (No EV) using multi fluidic resistive pulse sensing (MRPS) with the Spectradyne nCS1^™^ instrument. MRPS indicates that the majority of EVs and captured NEEVs were in the small EV size range. The average concentration of NEEVs was approximately 1.98 × 10^10^ particles per mL, which was approximately 30-fold higher than EV-depleted plasma (6.7 × 10^8^ particles per mL). The approximate concentration of EV used for NEEV enrichment was 9.45 × 10^10^ particles per mL. Note: Y-axis is log-transformed concentration (particles/mL).

Figure 1 shows the results for EV and NEEV characterization following MISEV2018 guidelines^57^. Flow cytometry results (Fig. 1A) showed that NEEVs were positive for EV marker CD63-FITC and NEEV marker CD171-APC compared to EV-negative control, beads, or unstained samples. Western blot analysis (Fig. 1B) showed that: (1) CD171 marker was enriched in NEEVs; (2) EV surface marker CD81 and EV internal marker Alix were present in EVs and NEEVs; and (3) EV negative marker Calnexin was not observed in EV or NEEV samples (note: the image displayed in Fig. 1B is from the same gel without cutting prior to antibody hybridizations; see Supplemental Figures S1-S4 for images of full-length gels). Transmission electron microscopy images of EVs and NEEVs (Fig. 1C) showed that the majority of EVs and captured NEEVs were in the small EV size range (Fig. 1D). The average concentration of NEEVs was approximately 1.98 × 10^10^ particles per mL, which was approximately 30-fold higher than EV-depleted plasma (6.7 × 10^8^ particles per mL). The approximate concentration of EV used for NEEV enrichment was 9.45 × 10^10^ particles per mL.

### 3.3 NEEV miRNA results

**Figure 2:**
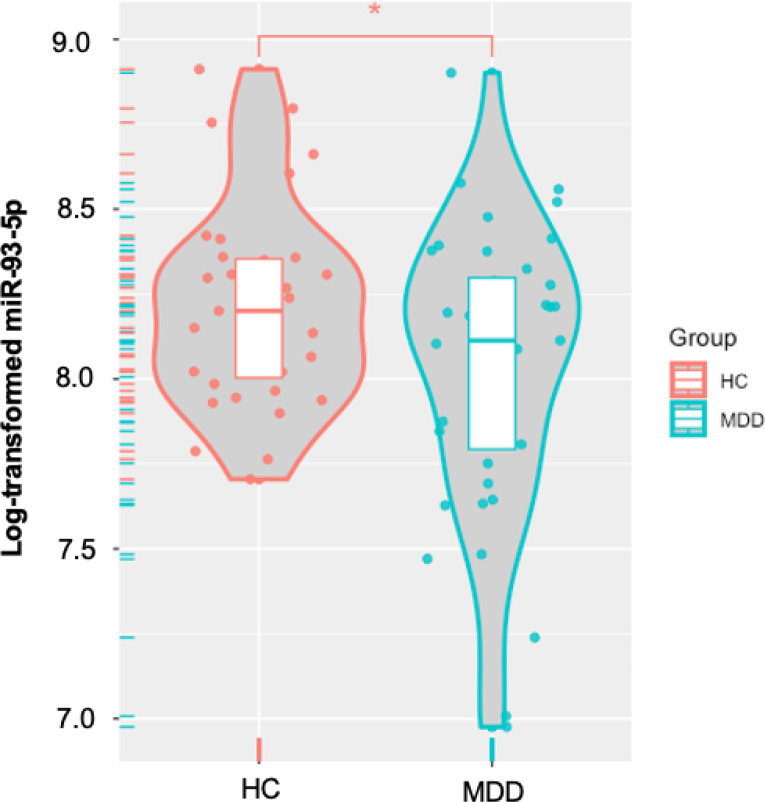
Differential expression of neuronal-enriched extracellular vesicle microRNA-93–5p (miR-93–5p) between MDD and HC groups. MDD = major depressive disorder. HC = healthy comparisons. * Indicates that the MDD group exhibited significantly lower levels of NEEV miR-93–5p expression than HC (*p* < 0.05).

The neuronal cell-specific marker miR-9 was expressed at a 15-fold higher rate in NEEV than EV (Supplemental Figure S5). One outlier from the MDD group was excluded for miR-93 analysis. MDD exhibited significantly lower levels of NEEV miR-93 expression than HC (*p* = 0.037, *d* = 0.482) (Fig. 2), a difference that remained after controlling for BMI (*p* = 0.035). In addition, miR-93 expression did not differ between unmedicated and SSRI-medicated MDD individuals (*p* = 0.398).

### 3.4 Immunoassay results

**Figure 3:**
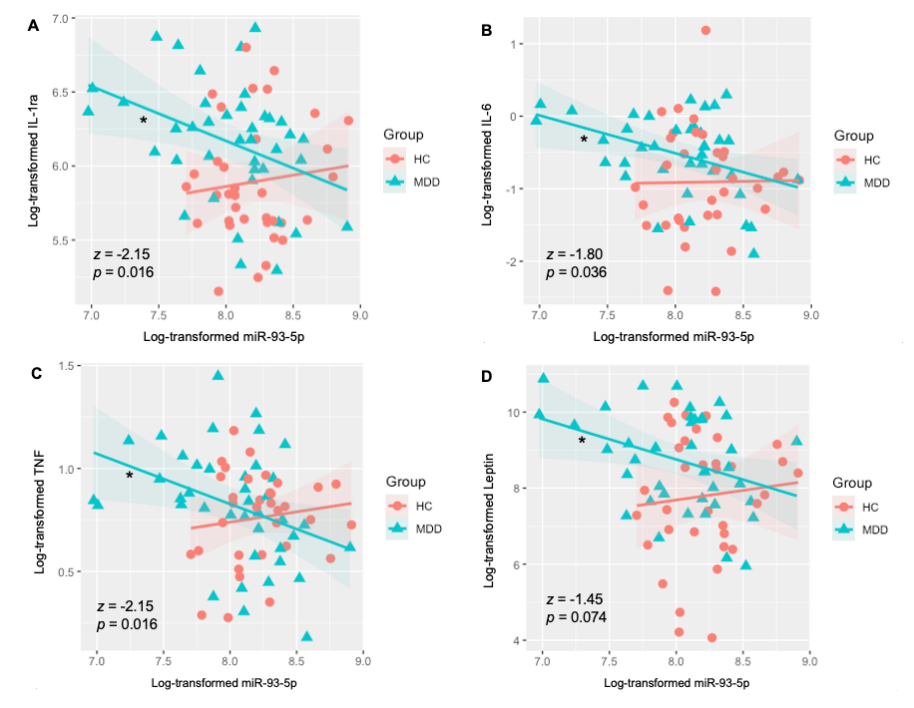
Differential associations between neuronal-enriched extracellular vesicle miR-93 and serum IL-1ra, IL-6, TNF and leptin, in the MDD and HC groups. MDD = major depressive disorder. HC = health comparisons. IL-1ra = interleukin-1 receptor antagonist. IL-6 = interleukin-6. TNF = tumor necrosis factor. *z*, *p*, Fisher’s r-to-z transformations (MDD>HC). * indicates a significant correlation within the MDD group.

MDD group exhibited significant higher levels of IL-1ra (*p* = 0.006, *d* = 0.657 ), IL-6 (*p* = 0.014, *d* = 0.596), CRP (*wilcox p* < 0.001, *d* = 1.186), and leptin (*wilcox p* = 0.021, *d* = 0.637) than HC. Group differences were observed in the slope of the relationship between NEEV miR-93 and serum IL-1ra (F_(1, 72)_ = 5.71, p = 0.020); serum IL-6 (F_(1, 70)_ = 5.66, p = 0.020); serum TNF (F_(1, 71)_ = 4.63, p = 0.035); and serum leptin (F_(1, 72)_ = 5.27, p = 0.025). Lower NEEV miR-93 expressions were associated with higher serum IL-1ra (*r* = −0.39, *p* = 0.013; Fig. 3A), IL-6 (*r* = −0.40, *p* = 0.012; Fig. 3B), TNF (*r* = −0.37, *p* = 0.018; Fig. 3C), and leptin (*r* = −0.34, *p* = 0.035; Fig. 3D) concentrations in MDD participants but no such relationship was observed in HCs (IL-1ra, *r* = 0.11, *p* = 0.528; IL-6, r = 0.01, p = 0.942; TNF, *r* = 0.13, *p* = 457; leptin *r* = 0.00, *p* = 0.996). No significant correlations were observed between NEEV miR-93 and serum CRP concentrations. Fisher’s r-to-z transformations indicated that the relationship between miR-93 expression and inflammatory/metabolic markers was significantly more negative in MDD than HC for IL-1ra (*z* = −2.15, *p* = 0.016), IL-6 (*z* = −1.80, *p* = 0.036), and TNF (*z* = −2.15, *p* = 0.016), and trending more negative in MDD than HC for leptin (*z* = −1.45, *p* = 0.074).

### 3.5 Neuroimaging results

**Figure 4:**
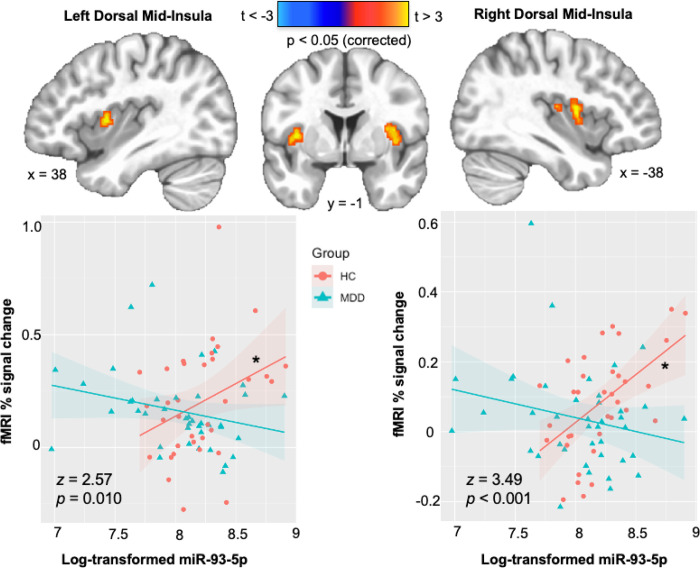
Differential association between neuronal-enriched extracellular vesicle miR-93 and interoception versus exteroception contrast during the interoceptive awareness task in the MDD and HC groups. MDD = major depressive disorder. HC = healthy comparisons. *Z, p*, Fisher’s r-to-z transformations (MDD>HC). * Indicates a significant correlation within the HC group (*p* < 0.05).

There were no significant group activation differences observed outside of the insular cortex, and thus, our analysis focused on clusters of observed insular activation. Specifically, group differences were observed in the slope of the relationship between NEEV mR-93 and the interoception versus exteroception contrast within the left (*F*_1,71_ = 6.34, *p*_corrected_ = 0.014) and right (*F*_1,71_ = 9.75, *p*_corrected_ = 0.006) dorsal mid-insula. Within the HC group, higher miR-93 expressions were associated with higher BOLD signal for the interoception versus exteroception contrast within the left (*r* = 0.34, *p*_corrected_ = 0.047) and right (*r* = 0.54, *p*_corrected_ = 0.002) dorsal mid-insula, but no such relationship was observed in MDD participants (Fig. 4). Fisher’s r-to-z transformations were applied to this correlation for each group and then compared; the results indicated that the relationship between miR-93 expression and interoception was significantly more positive in HC than MDD for the left (*z* = 2.57, *p* = 0.010) and right (*z* = 3.49, *p* < 0.001) dorsal mid-insula.

## 4. Discussion

This study aimed to elucidate the molecular processes underlying previously described mid-insula dysfunction during interoceptive processing in depression using brain NEEV measurement, serum markers of inflammation and metabolism, and whole brain fMRI recording. There were three main findings. Firstly, miR-93 expression in NEEVs was significantly diminished in individuals with Major Depressive Disorder (MDD) compared to Healthy Controls (HC). Secondly, a unique association emerged in MDD participants, where reduced miR-93 expression in NEEVs correlated with elevated serum concentrations of IL-1ra, IL-6, TNF, and leptin-establishing a connection between miR-93 expression in MDD and heightened inflammation. Lastly, in HC participants, but not in those with MDD, miR-93 expression in NEEVs exhibited a positive correlation with BOLD signals in the left and right dorsal mid-insula during interoception, linking miR-93 regulation to adaptive interoceptive processing in healthy individuals. Taken together, while healthy individuals demonstrate increased responsiveness to stress-induced epigenetic regulation of insular function during interoceptive processing, MDD participants exhibit a failure to do so. This highlights the potential role of insufficient miR-93 signaling and its altered relationship with systems-level interoceptive processing in contributing to interoceptive processing abnormalities in MDD. The pathways unveiled in this study could offer novel therapeutic targets for rectifying interoceptive dysfunction among individuals suffering from depression.

MiR-93 expression was lower in individuals with MDD than those without MDD. To better understand the role of NEEV miR-93 in different neuronal processes, we performed a biological pathway analysis with miRWalk^58^ by target mining the full mature miRNA, hsa-miR-93–5p, with miRBaseID. Several genes and biological pathways were identified after filtering with TargetScan, miRDB, and miRTarBase. Several differentially expressed genes were used during Gene Set Enrichment Analysis (GSEA), which identified 210 enriched genes, and 23 (out of 53) biological pathways that were significant, to include pathways centered on calcium ion transport, memory, and protein ubiquitination (**See Supplemental Table S1**). The pathways mentioned above are known to play a role in depression; for instance, the lack of ubiquitination of certain proteins^59–61^, memory disruption^62, 63^, and calcium ion signaling (linked to neuronal excitability and neurotransmitter release)^64, 65^, have all been linked to depression. These targets and more may be of interest or offer plausible explanations to the decreased interoceptive signaling found in depressed individuals, given that miR-93 in NEEV is attenuated.

We observed lower NEEV miR-93 associated with higher serum concentrations of inflammatory and metabolic markers, IL-1ra, IL-6, TNF, and leptin within MDD. Expanse literature shows that a subset of depressed individuals exhibits increased levels of pro-inflammatory cytokines, as those mentioned here^66–68^. The decreased expression of NEEV miR-93 in MDD, which was not seen in HC, may point to a possible mechanism of elevated inflammation in MDD. This in part may be due to the negative regulation of miR-93 on the interleukin receptor associated kinase-4 (IRAK-4), and in turn, suppression of inflammatory cytokines^69, 70^, posing a possible target for inflammation-associated depression. Some studies have showed that elevated leptin concentrations and leptin resistance are linked to depressed-related appetite increase or atypical feathers in MDD^14, 71, 72^. In the dataset involved in current analysis, we did observe higher serum leptin concentrations in MDD subjects than HC. The negative association between NEEV miR-93 expression and serum leptin concentration in MDD provided a possible treatment target for MDD with leptin-related metabolic dysfunctions.

We did not observe an association between miR-93 expression and interoceptive signaling in the brains of individuals with MDD, but we did find a relationship between NEEV miR-93 and higher interoception-associated insula activity in healthy individuals. This could suggest a homeostatic role of miR-93 from NEEV during intact interoception, although the precise nature of this relationship is unclear. Consistent with empirical and theoretical findings implicating the role of insular activity in subjective interoceptive and emotional states^73–75^, our observation of associations between miR-93 and VIA BOLD signal in left and right dorsal mid-insula might be interpreted to suggest a mechanism whereby the trafficking or regulation of NEEV miR-93 activity is intact and involved in interoceptive processing in healthy individuals, but dysfunctional in depressed individuals. However, this is a speculative notion and warrants further study, particularly with respect to longitudinal assays of the relationship between affective states, NEEV miR-93 activity, and neural indicators of interoception in depression.

Interoception is a process allowing individuals to continuously sense and integrate numerous visceral, physiological signals including autonomic and nociceptive input, emotional stimuli, hunger signals, and sleep, which are then perceived by the brain during continuous feedback^76^. miR-93–5p regulates many diverse gene products that influence a range of potentially associated processes ranging from inflammation to epigenetic modulation (**See Supplemental Figure S6**). It seems plausible that its expression is both a consequence of environmental exposure with long-term consequences, e.g. early life stress^77^, or subtle inflammatory processes^78^ that have been implicated in the pathophysiology of depression. Chronic stress, which is a risk factor for MDD, has been proposed to lead to increased in inflammation^77^, which in turn disrupt neural circuits involved in interoceptive processing^16^. Additionally, cytokines can affect the function of neurons and glia in the brain, leading to altered neural activity and connectivity^79^. Therefore, the association between lower miR-93 expression and higher serum concentrations of IL-1ra, IL-6, TNF, and leptin in individuals with MDD, might be interpreted to suggest that a low miR-93 expression level fails to regulate inflammatory cytokines in MDD. Again, further studies are needed to validate and extend this notion.

### Strengths.

We focused on evaluating how a neuronal process could be associated with interoceptive signaling using the innovative technique of NEEV isolation. For this, NEEV were isolated with the use of the transmembrane L1 cell adhesion molecule (L1CAM/CD171). A recent review article reported concern about using L1CAM/CD171 enriched EVs^80^, due the expression of L1CAM in other tissues of the body. We took careful steps to characterize and verify NEEVs following the MISEV2018 guidelines^57^, including: (1) flow cytometry of CD63 and CD171; (2) western blot analysis of EV surface markers, CD171 and CD81, EV internal marker – Alix, and the EV negative marker – calnexin; (3) EV and NEEV particle size and concentration measurements using the MRPS technology; (4) transmission electron microscopy imaging of EV and NEEV; (5) EV-depleted samples used as negative controls, and (6) the use of thrombin treatment for removal of fibrinogen from plasma, which potentially affects plasma EV separation and characterization. Additionally, data confirmed that the neuronal cell-specific marker miR-9 was expressed at a much higher rate in NEEV than EV, supporting the enriched neuronal origin of NEEV^56^.

### Limitations.

While this study revealed new insight into the possible role of NEEV miR-93 in interoception, there are several limitations. First, more than half of MDD patients were taking SSRIs, raising the possibility of serotonergic influences on the results. We believe this is unlikely as there was no difference on the VIA task or NEEV miR-93 expression between unmedicated MDD and SSRI-medicated individuals. Future work may address this by repeating the study in unmedicated participants only. Second, other unmeasured factors could have potentially affected our results, such as other types of medication, genetics, diet, and socioeconomic status. Third, although we characterized the neuronal enrichment of NEEV, we were unable to further subdivide the NEEV populations to determine neuron type. Future studies should be undertaken to replicate these findings, refine the observed relationships to specific neuronal subtypes, and longitudinally evaluate the degree to which NEEV signaling fluctuates with interoceptive and affective changes, reflecting the varied emotional landscape of depression.

## 5. Conclusions

This study suggests that MDD is associated with lower NEEV miR-93 expression, which may lead to interoceptive processing dysfunctions through altered epigenetic modulation of insular function, whereas healthy individuals may be more reactive to stress-induced regulation of miR-93 expression during interoceptive processing. The combination of neuroimaging and brain-enriched extracellular vesicle approaches provides an exciting opportunity to discover novel cellular disease targets for depression.

## Figures and Tables

**Table 1:** Sample Demographics and Clinical Characteristics

Group	MDD (*n* = 41) *Mean (sd)*	HC (*n* = 35) *Mean (sd)*	*p*-value
Age	34.22 (11.63)	30.03 (9.85)	.10^a^
Sex = Male (%)	11 (26.8)	14 (40.0)	.33^b^
IPAQ Category (%)			.01^b^
HEPA Active	10 (20.4)	20 (58.8)	
Minimally Active	11 (26.8)	8 (23.5)	
Inactive	20 (48.8)	6 (17.6)	
IPAQ MET-minutes per week	3849.07 (4279.15)	4659.21 (3678.56)	.01^a^
Body Mass Index	30.44 (4.60)	26.59 (4.90)	< .01^a^
PROMIS Depression Score	63.20 (6.55)	42.49 (6.61)	< .01^a^

Note. MDD = major depressive disorder. HC = healthy control. IPAQ, International Physical Activity Questionnaire; PROMIS, Patient-Reported Outcomes Measurement Information System Depression Score.

a Two Sample *t*-test

b *χ*^2^ test.
